# Intracortical myelin in individuals with alcohol use disorder: An initial proof‐of‐concept study

**DOI:** 10.1002/brb3.2762

**Published:** 2022-09-14

**Authors:** Vanessa Morris, Nicholas Bock, Luciano Minuzzi, James MacKillop, Michael Amlung

**Affiliations:** ^1^ Peter Boris Center for Addictions Research McMaster University Hamilton Canada; ^2^ Department of Psychology York University Toronto Canada; ^3^ Department of Psychology, Neuroscience, and Behaviour McMaster University Hamilton Canada; ^4^ Department of Psychiatry and Behavioural Neurosciences McMaster University Hamilton Canada; ^5^ Cofrin Logan Center for Addiction Research and Treatment University of Kansas Lawrence Kansas USA; ^6^ Department of Applied Behavioral Science University of Kansas Lawrence Kansas USA; ^7^ Department of Psychology University of New Brunswick Saint John Saint John Canada

**Keywords:** addiction, alcohol, intracortical myelin, MRI

## Abstract

**Introduction:**

Disruption of cortical gray matter and white matter tracts are well‐established markers of alcohol use disorder (AUD), but less is known about whether similar differences are present in intracortical myelin (ICM, i.e., highly myelinated gray matter in deeper cortical layers). The goal of this study was to provide initial proof‐of‐concept for using an optimized structural magnetic resonance imaging (MRI) sequence to detect differences in ICM in individuals with AUD compared to control participants reporting drinking within recommended guidelines.

**Methods:**

This study used an optimized 3T MRI sequence for high intracortical contrast to examine ICM‐related MRI signal in 30 individuals with AUD and 33 healthy social drinkers. Surface‐based analytic techniques were used to quantify ICM‐related MRI signal in 20 bilateral a priori regions of interest based on prior cortical thickness studies, and exploratory vertex‐wise analyses were examined using Cohen's *d* effect size.

**Results:**

The global distribution of ICM‐related signal was largely comparable between groups. Region of interest analysis indicated that AUD group exhibited greater ICM‐related MRI signal in precuneus, ventromedial prefrontal cortex, posterior cingulate, middle anterior cingulate, middle/posterior insula, and dorsolateral prefrontal cortex (Cohen's *d*s = 0.50–0.75). Four regions (right precuneus, ventromedial prefrontal cortex, posterior cingulate and left dorsolateral prefrontal cortex) remained significant (*p* < .05) after covarying for smoking status.

**Conclusion:**

These findings provide initial evidence of ICM differences in a moderately sized sample of individuals with AUD compared to controls, although the inflation of type 1 error rate necessitates caution in drawing conclusions. Robustly establishing these differences in larger samples is necessary. The cross‐sectional design cannot address whether the observed differences predate AUD or are consequences of heavy alcohol consumption.

## Graphical abstract

This study provides initial proof‐of‐concept for an optimized structural magnetic resonance imaging sequence to detect differences in intracortical myelin (ICM) in people with alcohol use disorder compared to social drinkers. Surface‐based analytic techniques were used to quantify ICM in 20 regions of interest and across the entire cortex. The global distribution of ICM was largely comparable between groups, although the alcohol use disorder group exhibited greater ICM signal in regions of precuneus, prefrontal cortex, posterior cingulate, anterior cingulate, and insula.

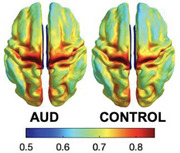

## 1 INTRODUCTION

Structural and functional disruptions of the cerebral cortex are well‐established indicators of alcohol use disorder (AUD) (Fritz et al., 2022; Sullivan et al., 2010). People who meet criteria for AUD have been found to exhibit thinner cerebral cortices (Durazzo et al., 2011; Fortier et al., 2011), smaller gray matter volume (Bühler & Mann, 2011), as well as smaller volume in the frontal cortex and other cortical and subcortical regions (Durazzo et al., 2011; Yang et al., 2016) relative to people without AUD. These neuroanatomical differences were confirmed in a mega‐analysis of previously published MRI data pooled from 23 laboratories including 3240 individuals (898 of whom were diagnosed with AUD) (Mackey et al., 2019). Of all substances examined (alcohol, nicotine, cocaine, methamphetamine, and cannabis), differences between AUD and control groups revealed the largest effect sizes for gray matter volume and cortical thickness in 27 cortical regions spanning frontal, temporal, parietal, and insular cortices. Other studies specifically focusing on cortical thickness in AUD have reported that decreased cortical thickness is related to a number of clinical indicators in AUD, including quantity/frequency of alcohol use and severity of alcohol‐related problems (Fortier et al., 2011; Mashhoon et al., 2014), poor treatment outcomes (Durazzo et al., 2011), and impaired inhibitory control (Pennington et al., 2015).

There is considerable interest in alcohol‐related myelin reductions as these may directly contribute to dysfunction in the central nervous system in people diagnosed with AUD. White matter is particularly susceptible to damage from alcohol and other drugs due to its low level of blood supply and the extensive energy requirements of oligodendrocyte function (Filley et al., 2017; Haroutunian et al., 2014). White matter atrophy and effects of alcohol on myelin are considered hallmarks of AUD‐related neuropathology (for a review, see Rice & Gu, 2019). Global deficits in white matter volume have been reported in numerous studies, as confirmed by a meta‐analysis of 19 studies with over 1300 participants (Monnig et al., 2013). A coordinate‐based meta‐analysis of 18 studies reported macro‐ and microstructural WM alterations in patients with AUD that were assigned to the genu and body of the corpus callosum, anterior and posterior cingulum, fornix, and the posterior limb of the internal capsule (Spindler et al., 2022). Evidence for disrupted myelin in AUD includes postmortem studies reporting demyelination in humans with a history of AUD and irregular folding and splitting of myelin membranes (Rice & Gu, 2019). Although primarily concentrated in the white matter, myelinated axons are also found in gray matter on local inhibitory interneurons and excitatory projection neurons in deep layers of the cortex (Nieuwenhuys, 2013; Rice & Gu, 2019). These deeper, myelinated layers of the cortex (layers IV‐VI) are commonly referred to as intracortical myelin (ICM). The functional role of ICM is not completely understood, though it is involved in synchronizing and speeding of neuronal signals through the cortex in support of cognitive processing. Decreased ICM in posterior cingulate cortex is correlated with decreased error processing and cognitive control in healthy individuals (Grydeland et al., 2015), and reduced ICM in insula and superior temporal gyrus is associated with increased performance variability (Grydeland et al., 2013). Grydeland et al. (2013) also found that developmental trajectories of ICM showed correspondence with other imaging indices of myelin structure (e.g., mean diffusivity on intracortical diffusion tensor imaging scans).

The extent to which ICM is altered in people with AUD remains unknown, although animal models suggest that ICM disruptions may accompany excessive alcohol exposure. Preclinical studies in rats have reported increased vulnerability of myelin in medial prefrontal cortex to neurotoxic effects of ethanol (Vargas et al., 2014). These differences may normalize following prolonged alcohol abstinence (Navarro & Mandyam, 2015). Medial prefrontal cortex is critical for higher order learning and social behavior and continues to develop into adulthood. In their review of myelin regulation in AUD, Rice and Gu (2019) conclude that animal studies show that alcohol‐mediated effects on myelination resemble some aspects of AUD in humans, and they emphasized that the extent to which myelinated gray matter is disrupted in AUD remains unknown and more extensive investigations are needed.

Advances in structural magnetic resonance imaging (MRI) have allowed for in vivo imaging of ICM content in human participants (Bock et al., 2013; Glasser & Van Essen, 2011). In the protocol developed by Bock et al. (2013), the T_1_‐weighted MRI contrast is optimized for differentiating cortical regions with low and high myelin content. This T_1_ image is commonly divided by a proton‐density‐weighted image to generate a ratio image that is strongly T_1_‐weighted with high intracortical contrast. Various studies have cross‐validated these MRI measurements of ICM with histological samples of nonhuman primate brain tissue (Bock et al., 2009), as well as postmortem human brain tissue (Fracasso et al., 2016). Together, these studies suggest that MRI‐generated ICM maps provide an accurate representation of underlying cortical myeloarchitecture.

The MRI protocol developed by Bock et al. (2013) has been used in studies of ICM in healthy participants (e.g., Rowley et al., 2015) and a limited number of studies of psychiatric disorders (Rowley et al., 2015; Sehmbi et al., 2019, 2018). These studies provide precedent for studying ICM in clinical disorders, but no neuroimaging studies to date have characterized ICM in addictive disorders. The ability to image ICM‐related signal in vivo affords a unique opportunity to explore effects of AUD in cortical subunits. This research may ultimately clarify the extent to which reductions in cortical thickness seen across studies are attributable to disruptions in ICM. To this end, the current study examined ICM in adults with AUD compared to social drinkers without AUD using the optimized ICM imaging protocol (Bock et al., 2013). The focus of this exploratory study was to provide initial proof‐of‐concept of the technique in an AUD sample. Based on extensive evidence that people with AUD show disruptions in myelination and animal models suggesting that myelin in gray matter is also disrupted by alcohol exposure (Rice & Gu, 2019), we hypothesized that participants meeting criteria for AUD would show disruption in ICM relative to participants who do not have AUD. Although a reduction in ICM is the most plausible disruption, we were hesitant to make a definitive prediction given the novelty of the ICM imaging approach and since all participants in this study were at minimum social drinkers. The latter point is important to consider because any amount of excessive alcohol use over prolonged periods of time could impact ICM as shown in studies of adult heavy drinkers (Topiwala et al., 2017).

## 2 METHODS

### 2.1 Participants

Participants were recruited from the general community and addiction treatment centers in Hamilton, Ontario, Canada. All participants were 25–55 years of age, had no MRI contraindications (e.g., metal in body), had no history of severe head trauma with loss of consciousness, neurological disorder, or severe psychiatric disorder (schizophrenia, bipolar disorder, posttraumatic stress disorder). Participants in the AUD group (*N* = 30) met DSM‐5 criteria for current AUD in the last 12 months and were excluded if they met DSM‐5 criteria for another substance use disorder other than nicotine. Participants in the social drinker (control) group (*N* = 33) reported weekly drinking which did not exceed NIAAA weekly drinking limits (i.e., <14/7 drinks per week for males/females) and did not meet DSM‐5 criteria for AUD or SUD (except nicotine). Sample characteristics are provided in Table 1. The Hamilton Integrated Research Ethics Board approved the study (Project #1747), and participants provided informed consent.

**TABLE 1:** Sample Characteristics

Variable	AUD (*N* = 30)M(SD), %, Median	CON (*N* = 33)M(SD), %, Median	*p*
Age	39.63 (9.65)	36.93 (10.46)	.29
Sex	33% female	58% female	.08
Race	86% white	84% white	.67
Education	14.43 (2.62)	17.45 (2.58)	<.001
Annual income	$30–45,000	$60–75,000	<.001
Weekly cannabis use	23%	6%	.01
Current smoker	50%	6%	<.001
FTND total (smokers only)	4.53 (2.67)	2.33 (2.08)	.20
Cigarettes/day (smokers only)	14.94 (8.59)	8.67 (7.09)	.25
DSM‐5 AUD severity	3% moderate 97% severe	N/A	
AUDIT	31.36 (5.50)	3.84 (1.75)	<.001
Drinks/week	16.06 (24.35)	3.06 (1.98)	<.01

Abbreviations: AUD, alcohol use disorder; CON, control; DSM‐5, Diagnostic and Statistical Manual of Mental Disorders, 5^th^ Edition; AUDIT, Alcohol Use Disorders Identification Test; FTND, Fagerstrom Test for Nicotine Dependence.

### 2.2 Procedures

Prospective participants completed an initial screening by telephone or web survey to determine eligibility. Eligible individuals were scheduled for an in‐person screening which included a battery of self‐report questionnaires, a clinical interview for AUD/SUD, and neurocognitive measures. Those who were confirmed as eligible were then scheduled for a 1‐h MRI scan at the Imaging Research Centre at St. Joseph's Healthcare Hamilton. All participants completed MRI safety screening prior to scanning. Participants received up to $50 in gift cards to local stores ($25 per session).

### 2.3 Measures

Participants completed several clinical and individual differences measures during the first session. A subset of these measures was analyzed in the current study, as detailed below.

#### 2.3.1 Structured Clinical Interview DSM‐5

Diagnoses of current (last 12 months) AUD and SUD were obtained via the Structured Clinical Interview for DSM‐5 (SCID‐5) (American Psychiatric Association, 2015). All participants completed the AUD module, and participants who reported using drugs also completed the SUD module for their most frequent substance.

#### 2.3.2 Alcohol‐related problems

The Alcohol Use Disorders Identification Test (AUDIT) (Saunders et al., 1993) was used as a self‐report measure of drinking behavior and alcohol‐related problems. The AUDIT is a 10‐item scale with total scores ranging from 0 to 40; scores of 8 or greater are indicative of hazardous alcohol drinking.

#### 2.3.3 Alcohol and substance use frequency

The timeline follow‐back interview was used to assess participants’ daily alcohol consumption for the past 30 days (Sobell & Sobell, 1992). However, given that 27 of 30 participants in the AUD group were recruited from treatment centers, there was a high percentage of participants who reported no alcohol consumption in the last 30 days. Thus, the drinks per week variable was for descriptive purposes only and not used in the primary analyses. The National Institute on Drug Abuse Alcohol, Smoking and Substance Involvement Screening Test (NIDA Modified ASSIST) (National Institute on Drug Abuse, n.d.) assessed frequency of use of 10 substances/categories (cannabis, cocaine, prescription stimulants, methamphetamine, inhalants, sedatives, hallucinogens, street opioids, and prescription opioids). Participants rated their use in the past 3 months, with responses ranging from never to multiple times daily.

#### 2.3.4 Nicotine dependence

Smoking status was assessed by asking participants if they currently smoked cigarettes (yes or no). Participants who endorsed current smoking completed the Fagerström Test for Nicotine Dependence (FTND; Heatherton et al., 1991), a validated self‐report assessment of cigarette consumption (e.g., cigarettes/day) and nicotine dependence severity.

#### 2.3.5 Demographics

A demographic questionnaire assessed age, sex assigned at birth, ethnicity, years of education, income, and handedness.

### 2.4 MRI scanning protocol

MRI images were acquired on a 3T General Electric Discovery system using a 32‐channel receive‐only radio frequency coil for the head and a transmit radio frequency body coil. All images were acquired with 1 mm isotropic resolution using a scan protocol developed by Bock et al.(2013). For the anatomical reference image for registration, we acquired a 3D T_1_‐weighted anatomical image of the whole head using a 3D inversion‐recovery gradient‐echo sequence (GE 3D BRAVO; Inversion time (TI) = 450 ms, TE = 3.2 ms, TR = 58.4 ms, flip angle = 12°, field of view (FOV) = 25.6 × 25.6 × 25.6 cm). ICM imaging included two T_1_‐weighted images with high intracortical contrast (one per hemisphere) using an inversion‐recovery gradient‐echo sequence (GE 3D BRAVO; TI = 1000 ms; TR = 8.4 ms; TE = 3.2 ms; flip angle = 12°; FOV = 24 × 10 × 24 cm; Matrix = 240 × 100 × 240 cm; TD = 1100 ms; centric phase encoding). Each hemisphere was imaged separately, and the resulting images were stitched together to form a complete anatomical image. Lastly, we collected a 3D proton density‐weighted image of the whole head (GE 3D FSPGR; TR = 7.9 ms, TE = 3.1 ms, flip angle = 4°, FOV = 24 × 17 × 24 cm) to normalize intensity inhomogeneities and remove T2*‐weighting in the high intracortical contrast T_1_‐weighted image. Total scanning time was 45 min.

### 2.5 Image preprocessing and data analysis

Image processing was performed predominately in FSL (v5.0.9), Freesurfer (v6.0.0), and the Human Connectome Project's Connectome Workbench software (v1.3.2). Preprocessing and ICM quantification closely followed published procedures (Glasser et al., 2013; Rowley et al., 2015; Sehmbi et al., 2019). Complete analysis code from preprocessing through group analysis steps can be downloaded from Open Science Framework (https://osf.io/pfwg6/). Raw DICOM image files were converted into NIFTI files using MRIcroGL's *dcm2nii*. To account for head motion between scans, the T_1_ with high intracortical contrast and proton‐density weighted images were registered to the reference T_1_‐weighted anatomical image using a six‐parameter linear affine registration using *FLIRT*. The T_1_‐weighted anatomical image was then linearly registered to the MNI 152 T_1_ 1 mm brain atlas and the corresponding transform applied to the other two images; this resulted in a single subject's images being in register with each other and each subject's images being in the same orientation as the MNI 152 atlas. The proton density‐weighted image was filtered using a 5 × 5 × 5 mm 3D median filter and a strongly T_1_‐weighted image (ratio) created by dividing the T_1_‐weighted high intracortical contrast image by the filtered proton‐density‐weighted image. This removed radiofrequency receive field (B_1_−) inhomogeneities, some radiofrequency transmit field (B_1_+) inhomogeneities, and T_2_*‐weighting arising from the gradient echo readout in the inversion‐recovery image. The ratio image still contained some B_1_+ inhomogeneity from the magnetization preparation portion of the inversion‐recovery pulse sequence. The T_1_‐weighted anatomical image was segmented in Freesurfer using *recon‐all*, with manual corrections for gray matter/pial boundary errors to yield surfaces corresponding to the cerebral‐spinal‐fluid/pial boundary (Freesurfer's lh.pial/rh.pial surfaces) and the pial/white matter boundary (lh.white/rh.white surfaces).

The remaining processing steps generated a surface at a middle depth in the cortex and mapped the intensity from the ratio image onto this surface to represent myelination in the cortex. They closely followed the Human Connectome Project's minimal processing pipeline using custom scripts for myelin mapping adapted from the HCP scripts (Glasser et al., 2013) (https://github.com/Washington‐University/HCPpipelines).^i^ These scripts used the lh.pial/rh/pial and lh.white/rh.white outputs of Freesurfer's *recon‐all* and the Connectome Workbench's *wb_command ‐surface‐average* to create a surface at a middle depth in the cortex between the pial and white matter surfaces. Each subject's middle depth surface was resampled into the HCP 32k_LR space (Van Essen et al., 2012) based on cortical folding attributes using *wb_command ‐surface‐resample*. This is important later for group analyses across all subjects. The image intensity from the ratio image representing myelin was mapped onto the middle depth surface using *wb_command ‐volume‐to‐surface‐mapping* with the *‐myelin‐style* option. Finally, surface data were brought into MATLAB using the *gifti* toolbox (https://www.artefact.tk/software/matlab/gifti/), and ICM surface visualizations were created using the *SurfStat* toolbox (http://www.math.mcgill.ca/keith/surfstat/).

**FIGURE 1:**
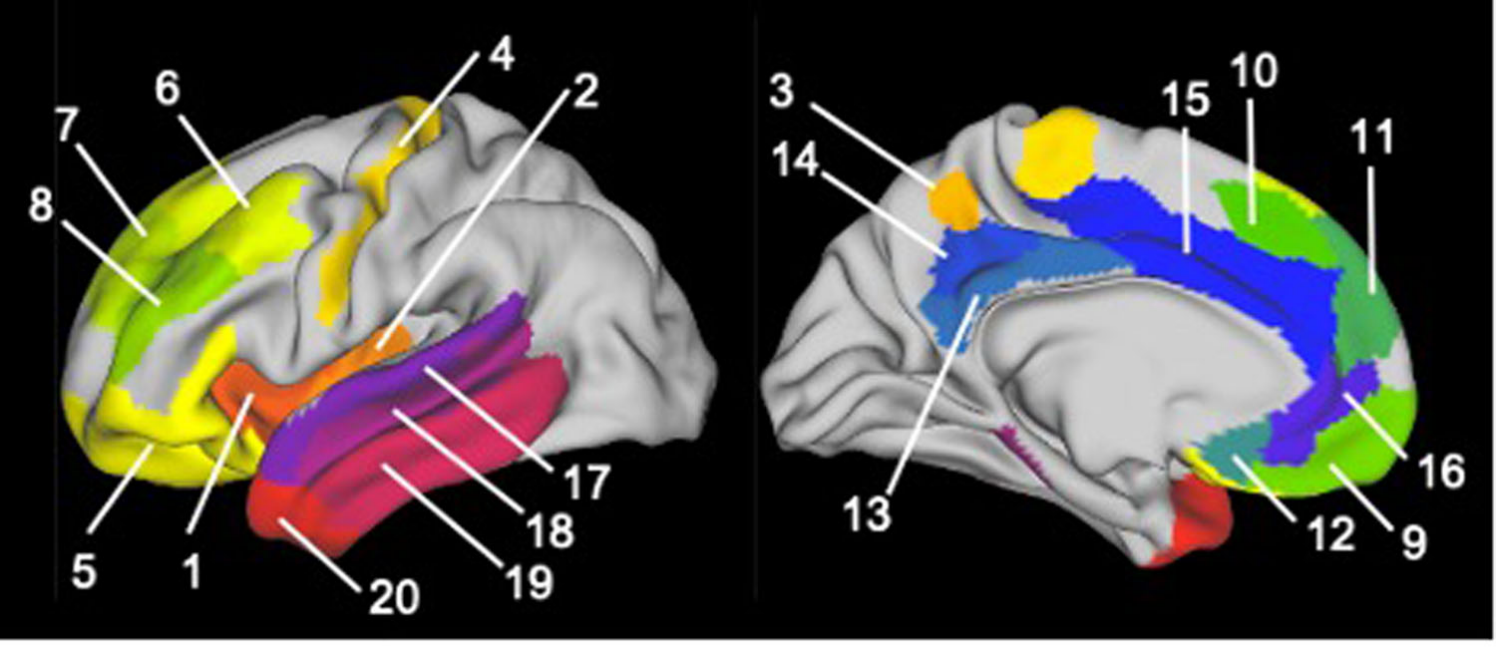
Anatomical locations of a priori regions of interest (Rgenerated from the multi‐modal parcellation atlas. Twenty ROIs were examined in each hemisphere. Region numbers correspond to numeric labels in Table 2.

Group comparisons were performed using an a priori anatomical region of interest (ROI) approach and a whole‐brain vertex‐wise analysis. ROIs were chosen based on theoretical models (Crews & Boettiger, 2009; Koob & Volkow, 2010; Longo et al., 2016), previous cortical thickness findings in AUD (Durazzo et al., 2011; Fortier et al., 2011; Lawyer et al., 2010; Mashhoon et al., 2014; Momenan et al., 2012; Morris et al., 2019), and studies of neurocognitive correlates of ICM (Grydeland et al., 2013, 2015). Specific ROIs were used from the multi‐modal parcellation atlas (MMP) (Glasser et al., 2017) in HCP 32k_LR space. We consolidated neighboring ROIs into composite regions corresponding to our a priori ROIs. The 20 bilateral ROIs examined are provided in Table 2, and anatomical locations are visualized in Figure 1.

**TABLE 2:** Regions of interest from multimodal parcellation atlas

No.	Region	MMP atlas region No.
1	Insula (anterior)	109,111,112
2	Insula (middle/posterior)	106,167,168
3	Precuneus	27
4	Primary motor cortex (M1)	8
5	Inferior frontal gyrus/orbitofrontal cortex (IFG)	66,75,76,77,90, 91,92,93,94,166
6	DLPFC (BA8)	67,68,70,73
7	DLPFC (BA9)	71,87
8	DLPFC (BA46)	83,84,85,86
9	Medial PFC (BA10)	65,88
10	Medial PFC (BA8)	63
11	Medial PFC (BA9)	69
12	Ventromedial PFC (VMPFC)	164
13	Posterior cingulate (PCC; BA23)	32,33,34
14	Posterior cingulate (PCC; BA31)	35,161,162
15	Middle cingulate	41,57,58,59,60, 62,179,180
16	Anterior cingulate (ACC)	61,64,165
17	Superior temporal (STG)	123,125,172,28
18	Middle temporal (MTG)	126,128,129,130,139,176
19	Inferior temporal (ITG)	132,133,134,137,177
20	Temporal pole	131,172

Abbreviations: DLPFC, dorsolateral prefrontal cortex; MMP, multimodal parcellation atlas; PFC, prefrontal cortex.

The mean ICM signal within each ROI was extracted and exported to SPSS v26 for group analyses using analysis of covariance (ANCOVA) models. Covariates were selected based on a two‐step process. Demographic and substance use variables that differed significantly between groups were subsequently examined in correlations with overall ICM‐related ratio signal (sum of 20 ROIs per hemisphere). Point‐biserial correlations were used for the binary smoking status and sex variables, Spearman rank‐order correlations were used for categorial variables (e.g., income, cannabis frequency), and Pearson correlations were used for continuous variables (age, years of education). Only variables that were significantly correlated (*p* < .05) were included as covariates. Exploratory whole‐brain analyses were conducted in two ways. First, we summed the ICM ratio signal values across all vertices in the complete set of 180 MMP atlas regions yielding a global measure of ICM across the entire cortex. Separate sums were generated for left and right hemispheres, and group differences in each hemisphere were examined using ANCOVA models. Second, we conducted an exploratory vertex‐wise analysis by calculating effect size (Cohen's *d*) for differences between AUD and control groups at each vertex. Since this is the first study to characterize ICM in AUD, we chose to be comprehensive and report statistical significance at *p* < .05. Anonymized imaging and self‐report data from this study are available upon request from the corresponding author.

## 3 RESULTS

### 3.1 Sample characteristics

The AUD group did not differ significantly from the control group with regard to sex, age, race, or handedness (Table 1), but the control group reported higher education and higher median income. As expected, the AUD group had a higher AUDIT score and drinks per week. Finally, a greater percentage of participants in the AUD group reported weekly use of cannabis (although no participants met criteria for cannabis use disorder) or being a current cigarette smoker. Neither cigarettes smoked per day nor FTND scores among smokers significantly differed between groups. Of the variables that differed between groups, none were significantly correlated with overall ICM (correlation coefficients < 0.15, *p*s > .05), except for a marginal association smoking status and right hemisphere ICM (*ρ* = .25, *p* = .053). Therefore, we conducted sensitivity analyses by repeating the primary analyses including smoking status as a covariate.

### 3.2 ICM group maps

**FIGURE 2:**
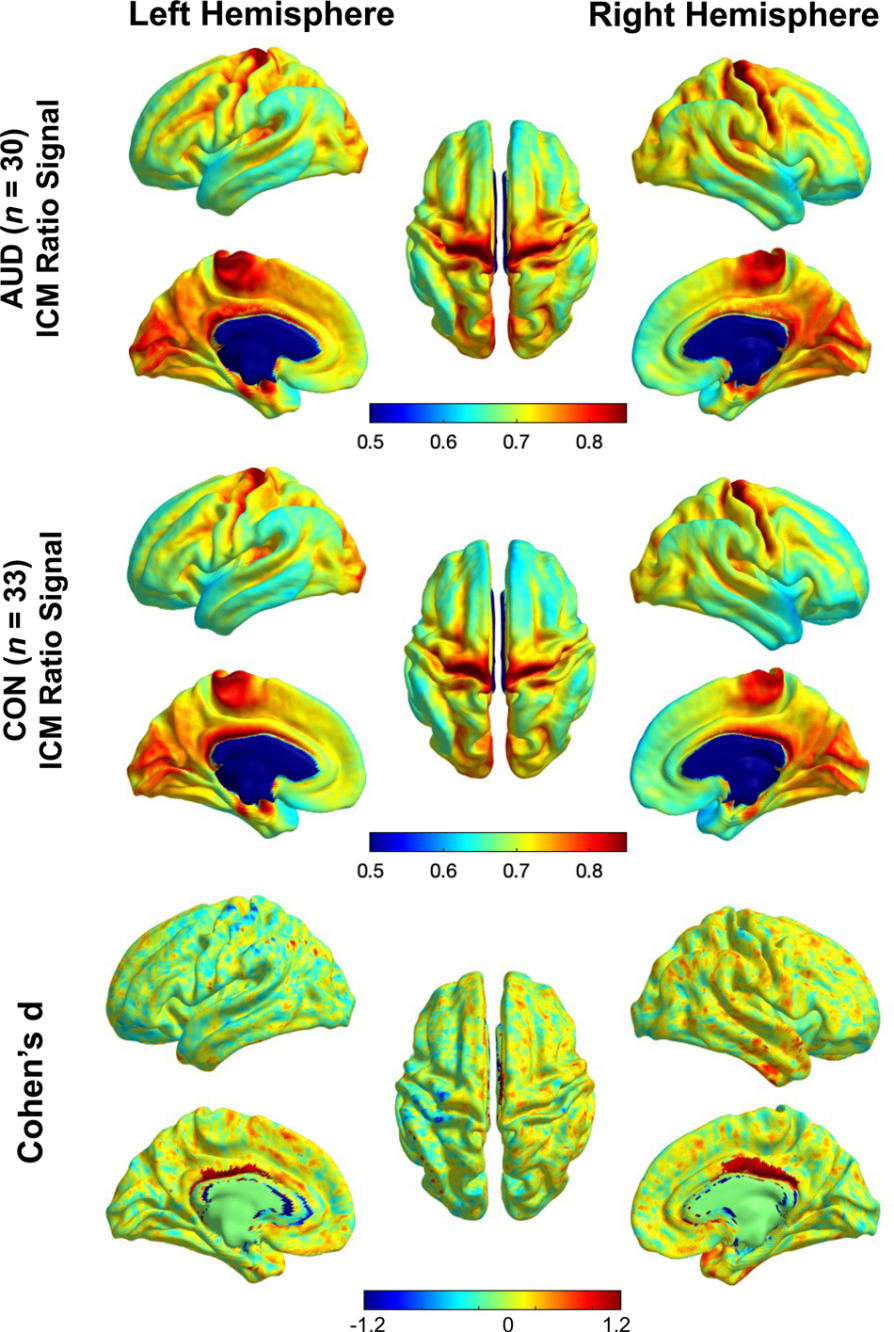
Group average intracortical myelin (ICM) maps for alcohol use disorder (AUD) (top) and control (middle) groups. Bottom panel depicts vertex‐wise effect size maps (Cohen's *d*) reflecting differences between AUD and controls. In each panel, data are projected onto the middle‐depth cortical surface, shown in lateral, medial, and superior views for left and right hemispheres separately.

Surface visualization maps depicting ICM‐related ratio signal for the AUD and the control groups are shown in Figure 2a–b. The cortical distribution of ICM‐related signal was generally similar across groups with the highest concentrations present in primary visual cortex, primary motor and primary sensory cortex, medial regions such as the anterior cingulate cortex (ACC), and the temporal pole. Visual comparison of the AUD and control maps suggested the possibility of greater ICM‐related MRI signal in the AUD group, particularly in medial frontal regions in the left hemisphere, the cingulate gyrus in both hemispheres, and the anterior temporal lobe in the right hemisphere.

### 3.3 Region of interest analysis

**FIGURE 3:**
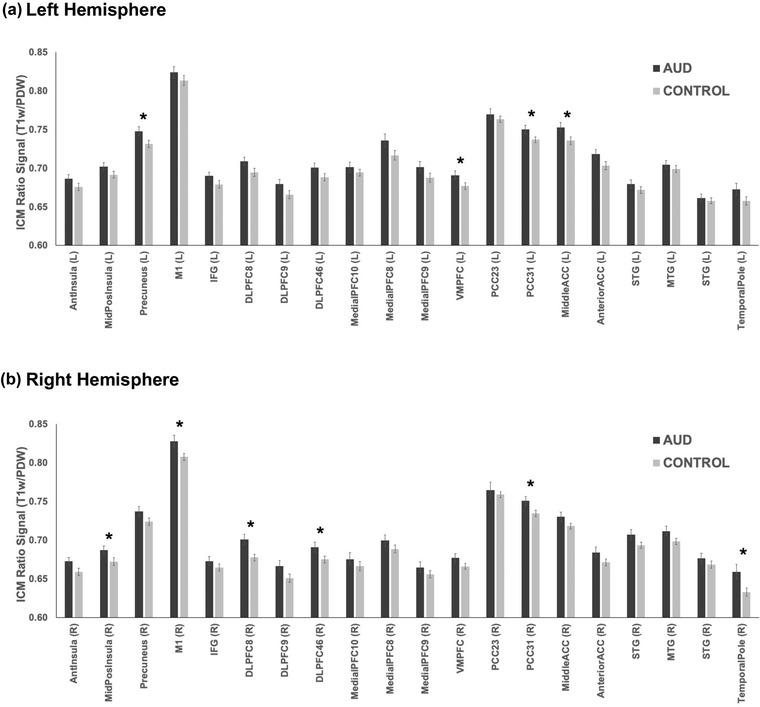
Extracted intracortical myelin (ICM)‐related ratio signal values for each a priori region of interest (ROI) for left hemisphere (Panel A) and right hemisphere (Panel B). Alcohol use disorder (AUD) group shown in black; control group shown in gray. Bars reflect mean +1 standard error. *p < .05. Region abbreviations are provided in Table 2.

Mean ICM‐related ratio signal was extracted from the a priori ROIs, and group differences were examined. A complete table of ICM values by group and brain region (including group means, mean differences, and 95% confidence intervals) is provided in Table S1. The AUD group exhibited greater ICM‐related ratio signal in 10 regions at *p* < .05 (uncorrected for multiple comparisons). See Figure 3 for ICM signal by group and Table 3 for statistical results. These included precuneus, ventromedial prefrontal cortex, posterior cingulate, and middle ACC in the left hemisphere; and middle/posterior insula, dorsolateral prefrontal cortex, posterior cingulate, temporal pole, and primary motor cortex in the right hemisphere. Effect sizes (Cohen's *d*) for these group differences ranged from 0.50 to 0.75, reflecting medium‐to‐large magnitude effects. When smoking was covaried, four of these effects remained significant (*p* < .05, uncorrected): precuneus, ventromedial prefrontal cortex and posterior cingulate in the left hemisphere and dorsolateral prefrontal cortex (BA8) in the right hemisphere. Importantly, none of these group differences remained significant after FDR correction.

**TABLE 3:** Region of interest analysis

		AUD vs. control				AUD vs. control	
		Left hemisphere				Right hemisphere	
No.	Region	*F*	*p*	*d*	*F*	*p*	*d*
1	Anterior insula	2.50	.12	0.40	3.83	.06	0.49
2	Mid‐posterior insula	2.32	.13	0.38	3.99	.05*	0.50
3	Precuneus	4.66	.04*	0.54	2.65	.11	0.41
4	Primary Motor (M1)	1.15	.29	0.27	5.59	.02*	0.59
5	IFG	2.56	.12	0.40	1.36	.25	0.29
6	DLPFC (BA8)	3.65	.06	0.48	8.88	.01*	0.75
7	DLPFC (BA9)	2.98	.09	0.43	3.36	.07	0.46
8	DLPFC (BA46)	3.16	.08	0.45	4.25	.04*	0.52
9	Medial PFC (BA10)	0.74	.39	0.21	0.80	.37	0.22
10	Medial PFC (BA8)	3.23	.08	0.45	1.57	.22	0.31
11	Medial PFC (BA9)	2.20	.14	0.37	1.22	.27	0.28
12	VMPFC	4.27	.04*	0.52	2.60	.11	0.40
13	PCC (BA23)	0.71	.40	0.21	0.26	.61	0.13
14	PCC (BA31)	4.02	.05*	0.50	5.28	.03*	0.58
15	Middle cingulate	4.27	.04*	0.52	3.09	.08	0.44
16	ACC	3.40	.07	0.46	2.63	.11	0.41
17	STG	1.28	.26	0.28	3.25	.08	0.45
18	MTG	0.55	.46	0.19	3.22	.08	0.45
19	ITG	0.36	.55	0.15	0.95	.33	0.24
20	Temporal pole	2.74	.10	0.41	6.01	.02*	0.61

*Note*: See Supporting Information for group means and 95% confidence intervals.

Abbreviations: ACC, anterior cingulate cortex; BA, Brodmann area; DLPFC, dorsolateral prefrontal cortex; IFG, inferior frontal gyrus; ITG, inferior temporal gyrus; MTG, middle temporal gyrus; PFC, prefrontal cortex; PCC, posterior cingulate cortex; STG, superior temporal gyrus; VMPFC, ventromedial prefrontal cortex.

* *p* < .05 uncorrected; ^**^ *p* < .01 uncorrected.

### 3.4 Exploratory whole‐brain analysis

Two exploratory analyses were conducted to examine differences in ICM across the entire cortex. First, we compared a total ICM index generated by summing ICM values across all vertices in the MMP atlas. Group means and 95% confidence intervals are presented in Table S2. There were no statistically significant differences between groups for the left hemisphere total ICM (*F*(1,61) = 1.95, *p* = .168, *d* = 0.41). For the right hemisphere, the difference between groups was marginally significant (*F*(1,61) = 3.73, *p* = .058, *d* = 0.46) potentially indicating greater total right hemisphere ICM for the AUD group compared to the control group. We also characterized group differences outside of the a priori ROIs by computing a whole‐brain vertex‐wise map of effect sizes (Cohen's *d*) for the differences between the AUD and control groups (see Figure 2c). Here, the largest effect sizes where AUD participants exhibited greater ICM signal compared to control participants were in anterior aspects of the inferior, middle, and superior temporal gyri, the bilateral ACC, and various medial and lateral frontal regions. With respect to regions showing less ICM signal in AUD compared to control, the largest effect sizes were found in left pre‐ and postcentral gyrus.

## 4 DISCUSSION

This study is the first to use an optimized MRI pulse sequence to characterize ICM in participants with AUD compared to social drinkers. Our results indicated that the overall cortical distribution of ICM appears to be similar between people with AUD and social drinkers although trend‐level group differences in right hemisphere total ICM may be indicative of greater ICM in AUD compared to control participants. When we conducted a finer‐grained ROI analysis, we found evidence of localized differences between groups with small‐to‐medium effect sizes. Comparing the two groups, we found greater ICM‐related ratio signal in 10 regions (uncorrected), including DLPFC, VMPFC, PCC, ACC, posterior insula, and precuneus, among others. When covarying for smoking status, differences in DLPFC, VMPFC, precuneus, and PCC remained significant. Taken together, the results of this study suggest there does not seem to be widespread differences in ICM between people with AUD and social drinkers, but some localized differences may exist. Although findings are preliminary, the most important contribution of this study is proof‐of‐concept for mapping ICM in vivo in AUD as a precursor to future studies in larger samples. Most notably, the inflation of type‐I error due to the large number of ROIs should be considered carefully as these effects may have been statistically significant by chance. A larger, more robust sample of people with AUDs and control participants is needed to confirm these results with greater certainty. Additional future directions are discussed below.

The direction of ICM differences was counter to our hypotheses. Based on prior imaging and postmortem studies with human participants and animal models of alcohol neurotoxicity, it is reasonable to expect that ICM would be reduced in AUD. Instead, we observed increased ICM in the AUD participants relative to controls in several regions. Unfortunately, the current study was not able to fully evaluate reasons for this result, and future research using these imaging techniques is necessary. Although speculative, one potential explanation for why the AUD participants had increased ICM signal relative to controls may be stem from myelin‐related compensatory mechanisms or other inflammatory responses to neurotoxic effects of chronic, excessive alcohol consumption. Alcohol has neurotoxic and inflammatory effects in both humans (Leclercq et al., 2017) and rodents (Pascual et al., 2014). As well, neuroinflammation can lead to the initiation of myelin repair (Glezer et al., 2007), and oligodendrocyte precursor cells may differentiate into remyelinating oligodendrocytes when demyelination is present in the case of an injury or lesion (Setzu et al., 2006). The biproducts of neuroinflammation—such as loss of tissue, demyelination, swelling—also can affect T_1_ relaxation times during MRI image acquisition (Albrecht et al., 2016) which may contribute to greater ICM signal in the AUD group. Importantly, since much of this work is in animal models, caution is needed when translating findings to humans. It would be informative for future imaging studies to examine changes in ICM while also collecting systemic and central markers of neuroinflammation (de Timary et al., 2017).

Comparing our results with previous ICM studies in healthy and clinical populations reveals some overlap in brain regions. We observed differences in regions of medial prefrontal cortex and anterior cingulate, which is consistent with preclinical and human studies reviewed in Rice and Gu (2019). The PCC region in the current study is in the same general location as the Grydeland et al.’s study of ICM in healthy participants that reported associations between ICM in PCC and error processing (Grydeland et al., 2015). The current study was not designed to test associations between ICM and neurocognitive performance; exploring whether increased ICM in PCC in people with AUD is related to neurocognitive performance is an important next step. Differences in DLPFC/Brodmann Area 46 have also been reported in previous studies with patients with bipolar disorder (e.g., Sehmbi et al., 2018), and DLPFC is consistently implicated in the neuropathology of AUD (e.g., Mackey et al., 2019).

Future studies of ICM in AUD should recruit a larger sample of participants with a wider range of alcohol involvement (e.g., across the continuum from social drinking through mild to severe AUD). As a proof‐of‐concept study, our intent was to compare a moderate/severe AUD group with social drinkers, but a dimensional study will help to further characterize ICM variation in relation to the full range of AUD severity. A larger sample size would also allow for examination of sex differences which was not feasible with the limited sample size in our study. Another important next step would be to observe ICM signal longitudinally. A longitudinal study could investigate when ICM‐related differences emerge and how alcohol consumption over time impacts ICM. This is important to address whether greater ICM in AUD results from excessive alcohol use over time or whether ICM differences are present prior to onset of AUD (i.e., a consequence or cause). A second priority is to examine what happens to ICM when people change their alcohol consumption in treatment and whether individual differences in ICM prospectively predict subsequent treatment outcomes (e.g., (Durazzo et al., 2011). Many of the AUD participants in the current study were actively engaged in treatment and were not currently drinking. Therefore, it is unclear whether the lack of significant group differences may have resulted from short‐term recovery of ICM in abstinence. Finally, it will be informative to examine ICM in conjunction with other imaging methods for assessing white matter structural integrity, such as intracortical diffusion tensor imaging (e.g., Grydeland et al., 2013). Doing so may provide a more comprehensive account of myelin disruption in AUD.

The current results should be considered in the context of the study's limitations. As noted above, the cross‐sectional design prevents testing of temporal or causal effects. The moderate sample size may have been underpowered to detect significant differences and precluded analyses of sex differences. The AUD and control groups also differed on several demographic and substance‐related variables, including education, income, cigarette smoking, and cannabis use. Of these variables, only current smoking was associated with ICM, and sensitivity analyses revealed that several differences remained even after controlling for smoking status. Finally, many participants in the AUD group were treatment‐seeking and were not currently drinking. Whether larger differences in ICM would emerge in participants who were not actively reducing their drinking is not known.

In conclusion, this is the first study to our knowledge to examine ICM‐related MRI signal in people meeting criteria for AUD. The distribution of ICM across the cortex was largely similar between the AUD and control groups, although a more precise analysis revealed several areas of potentially greater ICM signal in the AUD participants even after controlling for cigarette smoking. These initial findings offer proof‐of‐concept for studying ICM in addiction samples and provide a foundation for future studies to unpack the clinical and neurocognitive significance of the differences observed, neurobiological mechanisms (e.g., compensatory or neuroinflammatory changes), and potential for changes in ICM following treatment.

## CONFLICT OF INTEREST

James MacKillop is a principal in BEAM Diagnostics Inc., but no BEAM products were used in this study. No other authors have conflicts of interest to declare.

### PEER REVIEW

The peer review history for this article is available at https://publons.com/publon/10.1002/brb3.2762.

## Supporting information

Supplementary Table 1 Intracortical Myelin Ratio Signal by Group and Regions of Interest
Supplementary Table 2 Total Intracortical Myelin Signal by Group and Hemisphere

## Data Availability

Anonymized imaging and self‐report data from this study are available upon request from the corresponding author.

## NOTE

i Our processing scripts differed from the standard HCP pipeline scripts in three ways. First, the resolution of our data was 1.0 mm isotropic, not 0.7 mm isotropic. Second, our scanning protocol did not include a T_2_‐weighted image along with the T_1_‐weighted image that is used to improve cortical surface segmentation. Instead, we performed these corrections manually. Third, we are mapping our T_1_‐weighted ratio image instead of a T_1_ / T_2_ contrast.
