# Supplementary material for: Intracortical myelin in individuals with alcohol use disorder: An initial proof‐of‐concept study
Source: Brain Behav. 2022 Sep 14;12(10):e2762. doi: 10.1002/brb3.2762 (PMC9575605; doi:10.1002/brb3.2762)
Supplement: Supplementary file 1 — Supplementary Table 1 Intracortical Myelin Ratio Signal by Group and Regions of Interest Supplementary Table 2 Total Intracortical Myelin Signal by Group and Hemisphere [file BRB3-12-e2762-s001.docx]

**Supplementary Table 1**

Intracortical Myelin Ratio Signal by Group and Regions of Interest

|  |  | Left Hemisphere | | | |  | | Right Hemisphere | | | | |  |
| --- | --- | --- | --- | --- | --- | --- | --- | --- | --- | --- | --- | --- | --- |
| # | Region | *AUD M(SE)* | *CON M(SE)* | *M_Diff_* | *95% C.I.* | |  | | *AUD M(SE)* | *CON M(SE)* | *M_Diff_* | *95% C.I.* | |
| 1 | Anterior Insula | 0.687 (0.005) | 0.676 (0.005) | 0.011 | (-0.003, 0.024) | |  | | 0.673 (0.005) | 0.659 (0.005) | 0.014 | (0.000 0.027) | |
| 2 | Mid-Posterior Insula | 0.702 (0.005) | 0.692 (0.004) | 0.010 | (-0.003, 0.023) | |  | | 0.687 (0.006) | 0.672 (0.005) | 0.015 | (0.000, 0.029) | |
| 3 | Precuneus | 0.748 (0.006) | 0.732 (0.005) | 0.016 | (0.001, 0.031) | |  | | 0.737 (0.007) | 0.724 (0.004) | 0.013 | (-0.003, 0.029) | |
| 4 | Primary Motor (M1) | 0.824 (0.008) | 0.814 (0.006) | 0.010 | (-0.009, 0.030) | |  | | 0.828 (0.008) | 0.808 (0.004) | 0.020 | (0.003, 0.037) | |
| 5 | IFG | 0.690 (0.005) | 0.679 (0.005) | 0.011 | (-0.002, 0.025) | |  | | 0.673 (0.006) | 0.665 (0.005) | 0.008 | (-0.006, 0.023) | |
| 6 | DLPFC (BA8) | 0.709 (0.005) | 0.695 (0.005) | 0.015 | (0.000, 0.029) | |  | | 0.701 (0.007) | 0.678 (0.004) | 0.023 | (0.008, 0.038) | |
| 7 | DLPFC (BA9) | 0.680 (0.006) | 0.666 (0.005) | 0.014 | (-0.002, 0.030) | |  | | 0.667 (0.007) | 0.651 (0.005) | 0.016 | (-0.001, 0.033) | |
| 8 | DLPFC (BA46) | 0.701 (0.006) | 0.688 (0.005) | 0.013 | (-0.001, 0.027) | |  | | 0.691 (0.007) | 0.675 (0.004) | 0.016 | (0.001, 0.031) | |
| 9 | Medial PFC (BA10) | 0.701 (0.007) | 0.694 (0.004) | 0.007 | (-0.009, 0.023) | |  | | 0.676 (0.008) | 0.667 (0.006) | 0.009 | (-0.010, 0.028) | |
| 10 | Medial PFC (BA8) | 0.736 (0.008) | 0.717 (0.006) | 0.019 | (-0.002, 0.039) | |  | | 0.699 (0.007) | 0.689 (0.005) | 0.011 | (-0.006, 0.028) | |
| 11 | Medial PFC (BA9) | 0.702 (0.007) | 0.688 (0.006) | 0.014 | (-0.004, 0.031) | |  | | 0.665 (0.007) | 0.656 (0.005) | 0.009 | (-0.007, 0.025) | |
| 12 | VMPFC | 0.691 (0.006) | 0.677 (0.004) | 0.014 | (0.001, 0.028) | |  | | 0.677 (0.006) | 0.666 (0.004) | 0.011 | (-0.002, 0.024) | |
| 13 | PCC (BA23) | 0.770 (0.007) | 0.763 (0.004) | 0.007 | (-0.009, 0.022) | |  | | 0.764 (0.011) | 0.759 (0.004) | 0.006 | (-0.016, 0.027) | |
| 14 | PCC (BA31) | 0.750 (0.005) | 0.737 (0.004) | 0.013 | (0.000, 0.026) | |  | | 0.751 (0.006) | 0.735 (0.004) | 0.016 | (0.002, 0.030) | |
| 15 | Middle Cingulate | 0.753 (0.007) | 0.736 (0.005) | 0.017 | (0.001, 0.033) | |  | | 0.731 (0.006) | 0.718 (0.004) | 0.012 | (-0.001, 0.026) | |
| 16 | ACC | 0.718 (0.006) | 0.703 (0.005) | 0.015 | (-0.001, 0.031) | |  | | 0.684 (0.007) | 0.671 (0.004) | 0.013 | (-0.003, 0.028) | |
| 17 | STG | 0.680 (0.005) | 0.672 (0.004) | 0.008 | (-0.006, 0.021) | |  | | 0.707 (0.007) | 0.694 (0.004) | 0.014 | (-0.001, 0.028) | |
| 18 | MTG | 0.705 (0.005) | 0.699 (0.005) | 0.005 | (-0.009, 0.019) | |  | | 0.712 (0.006) | 0.699 (0.004) | 0.013 | (-0.001, 0.028) | |
| 19 | ITG | 0.662 (0.005) | 0.658 (0.004) | 0.004 | (-0.008, 0.016) | |  | | 0.676 (0.007) | 0.669 (0.004) | 0.008 | (-0.008, 0.024) | |
| 20 | Temporal Pole | 0.673 (0.008) | 0.658 (0.005) | 0.015 | (-0.003, 0.033) | |  | | 0.659 (0.010) | 0.633 (0.005) | 0.026 | (0.005, 0.047) | |

*Note*: AUD = alcohol use disorder; CON = control group; M = Mean; SE = Standard error; MDiff = mean difference; IFG = inferior frontal gyrus; DLPFC = dorsolateral prefrontal cortex; PFC = prefrontal cortex; BA = Brodmann area; VMPFC = ventromedial prefrontal cortex; PCC = posterior cingulate cortex; ACC = anterior cingulate cortex; STG = superior temporal gyrus; MTG = middle temporal gyrus; ITG = inferior temporal gyrus

**Supplementary Table 2**

Total Intracortical Myelin Signal by Group and Hemisphere

| Hemisphere | *AUD M(SE)* |  | *CON M(SE)* | *M_Diff_* | *95% C.I.* |
| --- | --- | --- | --- | --- | --- |
| Left Hemisphere Total ICM | 21431.28 (151.75) |  | 21170.84 (112.25) | 260.44 | (-56.01, 576.89) |
| Right Hemisphere Total ICM | 21368.10 (176.46) |  | 20973.89 (110.48) | 412.21 | (-31.81, 856.23) |

*Note:* AUD = alcohol use disorder; CON = control group; M = Mean; SE = Standard error; MDiff = mean difference
